# c-Jun *N*-Terminal Kinase Phosphorylation Is a Biomarker of Plitidepsin Activity

**DOI:** 10.3390/md11051677

**Published:** 2013-05-21

**Authors:** María J. Muñoz-Alonso, Enrique Álvarez, María José Guillén-Navarro, Marina Pollán, Pablo Avilés, Carlos M. Galmarini, Alberto Muñoz

**Affiliations:** 1 PharmaMar, Avda. De los Reyes, 1, Pol. Ind. La Mina-Norte, Colmenar Viejo E-28770, Madrid, Spain; E-Mails: tumorman@earthlink.net (E.A.); mjguillen@pharmamar.com (M.J.G.-N.); paviles@pharmamar.com (P.A.); cgalmarini@pharmamar.com (C.M.G.); 2 National Epidemiology Centre, Monforte de Lemos, 5, Madrid E-28029, Spain; E-Mail: mpollan@isciii.es; 3 Institute for Biomedical Research “Alberto Sols”, Spanish National Research Council (CSIC), Autonomous University of Madrid, Arturo Duperier, 4, Madrid E-28029, Spain; E-Mail: amunoz@iib.uam.es

**Keywords:** plitidepsin, Aplidin, JNK, biomarker, xenograft

## Abstract

Plitidepsin is an antitumor drug of marine origin currently in Phase III clinical trials in multiple myeloma. In cultured cells, plitidepsin induces cell cycle arrest or an acute apoptotic process in which sustained activation of c-Jun *N*-terminal kinase (JNK) plays a crucial role. With a view to optimizing clinical use of plitidepsin, we have therefore evaluated the possibility of using JNK activation as an *in vivo* biomarker of response. In this study, we show that administration of a single plitidepsin dose to mice xenografted with human cancer cells does indeed lead to increased phosphorylation of JNK in tumors at 4 to 12 h. By contrast, no changes were found in other *in vitro* plitidepsin targets such as the levels of phosphorylated-ERK, -p38MAPK or the protein p27^KIP1^. Interestingly, plitidepsin also increased JNK phosphorylation in spleens from xenografted mice showing similar kinetics to those seen in tumors, thereby suggesting that normal tissues might be useful for predicting drug activity. Furthermore, plitidepsin administration to rats at plasma concentrations comparable to those achievable in patients also increased JNK phosphorylation in peripheral mononuclear blood cells. These findings suggest that changes in JNK activity provide a reliable biomarker for plitidepsin activity and this could be useful for designing clinical trials and maximizing the efficacy of plitidepsin.

## 1. Introduction

Plitidepsin (Aplidin^®^, APL) is a marine cyclic depsipeptide originally isolated from the tunicate *Aplidium albicans* and currently obtained by synthesis. Plitidepsin is under Phase III clinical development in patients with relapsed/refractory multiple myeloma (ADMYRE trial).

Plitidepsin has demonstrated strong anticancer activity in a large variety of human cancer cell lines *in vitro* and in xenografted mice [1]. In cultured cells from solid tumors, plitidepsin induces dose-dependent cell cycle arrest or an acute apoptotic process due primarily to the sustained activation of c-Jun *N*-terminal kinase (JNK) as revealed by an increased level of phosphorylation [2,3]. Plitidepsin also activates other kinases such as the epidermal growth factor receptor, protein kinase C-δ and the extracellularly regulated and p38 mitogen-activated protein kinases (ERK, p38MAPK) in a cell-dependent context [4,5]. In breast cancer cells, plitidepsin induces several early response genes such as c-*JUN*, *JUNB*, *JUND*, c-*FOS*, *FOSB* and *FRA1*, as well as *RELA*/p65, the major component of the nuclear transcription factor *kappa* B (NF*k*B), while also decreasing the cellular content of the c-MYC protein [6]. Additionally, a series of candidate plitidepsin target genes have been identified in transcriptomic and proteomic studies in several cell types [7,8,9]. In sarcoma cells, plitidepsin increases the cellular content of the p27^KIP1^ cell cycle inhibitor [10]. 

Plitidepsin has strong activity against haematological cancer cells at nanomolar concentrations, in which it activates both the intrinsic/mitochondrial and extrinsic/death receptor apoptotic pathways [9,11,12,13]. Remarkably, plitidepsin-induced apoptosis is only partially dependent on caspases and independent of the *TP53* tumor suppressor gene status [5,11]. In haematological malignancies (multiple myeloma and leukemia cell lines), plitidepsin induces JNK activation and translocation from the cytosol to the plasma membrane lipid rafts [9,14].

The objective of this study was to identify a potential pharmacodynamic biomarker of plitidepsin. In view of the results obtained in a panel of haematological cancer cell lines, we have evaluated JNK phosphorylation as a potential biomarker of plitidepsin activity *in vivo*. The level of JNK phosphorylation was examined in the tumors from athymic mice xenografted with human leukemia K562 cells. In such K562 tumor-bearing mice, administration of a single plitidepsin dose resulted in increased phosphorylated-JNK levels in tumors at 4 to 12 h. A similar time course and increase in phosphorylated-JNK was also observed in the spleens of the host mice. Furthermore, intravenous administration of plitidepsin to rats significantly increased JNK phosphorylation in peripheral mononuclear blood cells eight hours after administration. In contrast, no changes were found in the levels of phosphorylated-ERK or -p38MAPK. Likewise, the expression of p27^KIP1^ was unaffected by plitidepsin. These results validate phosphorylated JNK as a pharmacodynamic biomarker of plitidepsin in xenografted tumors as well as in normal surrogate tissues.

## 2. Results and Discussion

### 2.1. Plitidepsin Induces JNK Phosphorylation in Cultured K562 Leukemia and Other Hematological Cancer Cells

Previous studies have shown that plitidepsin induces JNK phosphorylation in a variety of cultured solid and hematological cancer cell lines [3,4,5,9,11,15]. Given the advanced clinical development of plitidepsin in multiple myeloma and leukemia, we have evaluated the effect of plitidepsin on the viability and the level of JNK phosphorylation in a panel of these different cancer cell types.

**Figure 1:**
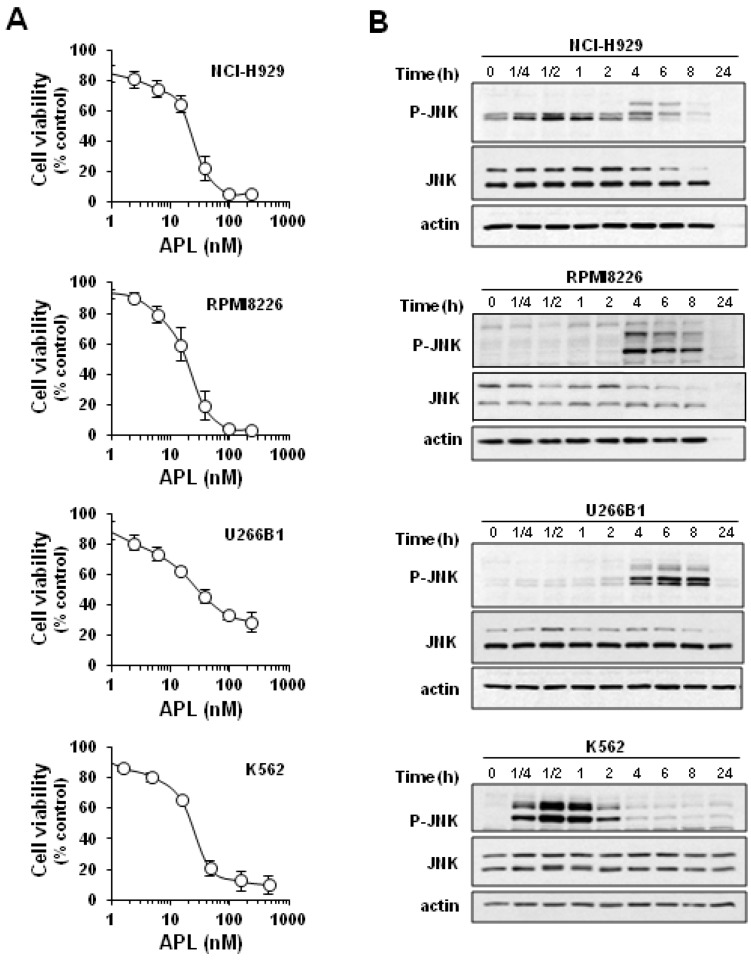
Plitidepsin decreases viability and induces c-Jun *N*-terminal kinase (JNK) activation in human hematological cancer cells. (**A**) Multiple myeloma NCI-H929, RPMI8226 and U266B1 cells, and leukemic K562 cells were incubated in the presence of the indicated concentrations of plitidepsin (APL) for 24 h and cell viability was determined by the MTT assay. Data are mean ± SEM obtained in three experiments performed in quadruplicate. (**B**) Western blot analysis showed the levels of total JNK and phosphorylated JNK (p-JNK) protein in cells incubated with plitidepsin (100 nM) for the indicated times. Actin was used as loading control.

Plitidepsin showed a strong, concentration-dependent effect on the viability of multiple myeloma NCI-H929, RPMI8226 and U266B1 cells (IC_50_ of ~11, 13 and 34 nM at 24 h post-treatment) and of chronic myelogenous leukemia K562 cells (IC_50_ of ~20 nM) (Figure 1A). Western blot analysis revealed that plitidepsin causes a progressive increase in the level of phosphorylated JNK in the multiple myeloma cell lines, which persists until cell death (Figure 1B). In K562 cells, plitidepsin also induced JNK phosphorylation that was extremely rapid (within the first 15 min of treatment) and sustained, reaching a maximum at 30 to 60 min (Figure 1B). These results suggest that K562 cells are good responders to plitidepsin cytotoxicity and JNK activation and hence provide a suitable model for assessing JNK phosphorylation as a potential biomarker of plitidepsin in xenografted mice. An additional reason for choosing K562 cells for *in vivo* studies is their expression of the chimeric BCR-ABL protein, which allows the purity of the tumor and host tissue samples to be controlled. 

To optimize the detection of phosphorylated JNK in tissue extracts, immunoprecipitation was used to examine the specificity of two widely used commercial anti-phospho-JNK antibodies. As shown in Supplementary Figure S1, although both antibodies recognized phosphorylated JNK in these assays, the anti-p-JNK sc-6254 antibody from Santa Cruz that rendered a better signal in whole protein extracts was selected for the *in vivo* study.

### 2.2. Plitidepsin Induces JNK Phosphorylation in Both Tumors and Spleens from K562 Xenografts

To evaluate the ability of plitidepsin to active JNK *in vivo* we first subcutaneously implanted K562 cells in athymic nude mice (5 × 10^6^ cells/mouse). Twenty days after initial tumor detection, mice were randomized into treatment and control groups (five mice/group) and treated animals received plitidepsin at 200 μg/kg. Mice in the control group were sacrificed immediately after plitidepsin administration (0 h). Tumors and spleens of all animals were harvested at several time points after treatment.

**Figure 2:**
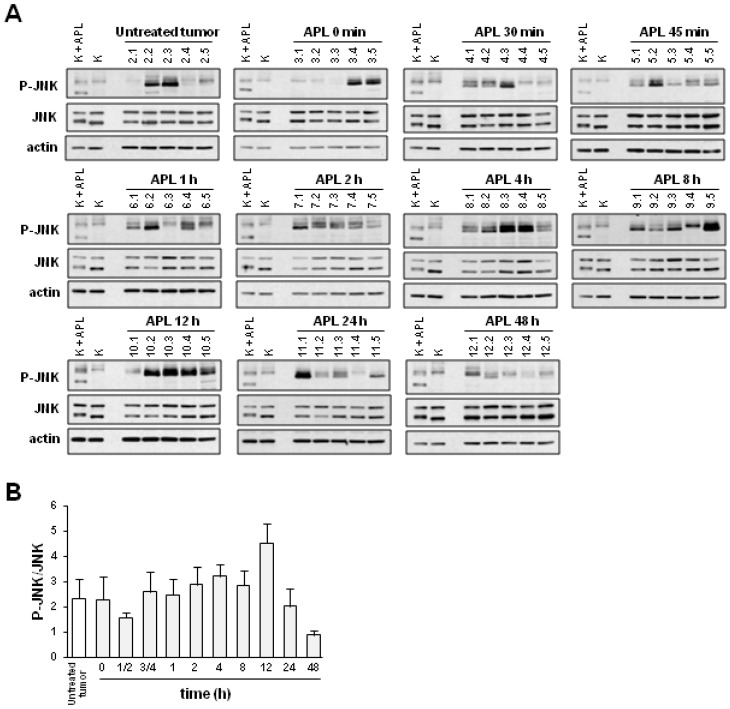
Plitidepsin increases the level of phosphorylated JNK in tumors of K562-bearing mice. Mice xenografted with K562 cells received a single administration of plitidepsin (APL; 200 μg/kg) and either immediately (0 min) or at the indicated times after injection were sacrificed (5 mice/group). (**A**) Western blot analysis of total (JNK) and phosphorylated JNK (p-JNK) in excised tumors. Actin was used as loading control. K, K562 cells; K + APL, plitidepsin-treated K562 cells. (**B**) Quantification of p-JNK levels (normalized to those of total JNK) and related to that of vehicle-treated K562 sample at the indicated times after APL administration. Mean ± SEM values of each group of animals are shown.

Western blot analysis of JNK phosphorylation in xenografted K562 tumors showed substantial variability within each group of animals; notwithstanding, between 4 and 12 h after plitidepsin treatment, a clear increase is seen in the number of animals with high levels of phosphorylated JNK (Figure 2A). No changes were observed in the level of total JNK protein. Samples from plitidepsin- or vehicle-treated K562 cells were included in each gel in order to compare the signals obtained in the different membranes. Quantification of phosphorylated JNK upon normalization to total JNK and to differences in inter-membrane signals was performed by optical densitometry (Figure 2B). A tendency for an increased level of phosphorylated JNK was observed in tumors corresponding to 4 to 12 h after plitidepsin administration. This effect was transient, as JNK phosphorylation returned to pretreatment levels at 24–48 h after dosing.

Since *in vitro*, the phosphorylation of p38MAPK and ERK and the expression level of the p27^KIP1^ protein is known to increase in response to plitidepsin in several cultured cell lines [4,5,10], we also examined the *in vivo* tissues for the activation of these kinases and the level of p27^KIP1^ using appropriate antibodies. In contrast to the *in vitro* data, plitidepsin did not change the level of phosphorylated-ERK or -p38MAPK in xenografted K562 tumors (Supplementary Figure S2). This result discards these kinases as markers of plitidepsin action *in vivo*. Likewise, no changes were found in the level of p27^KIP1^ expression following plitidepsin administration (Supplementary Figure S3). The purity of the excised tumors were confirmed by the high levels of the fusion BCR-ABL protein, expressed in the xenografted K562 cells, found in the tumor samples (Supplementary Figure S4).

**Figure 3:**
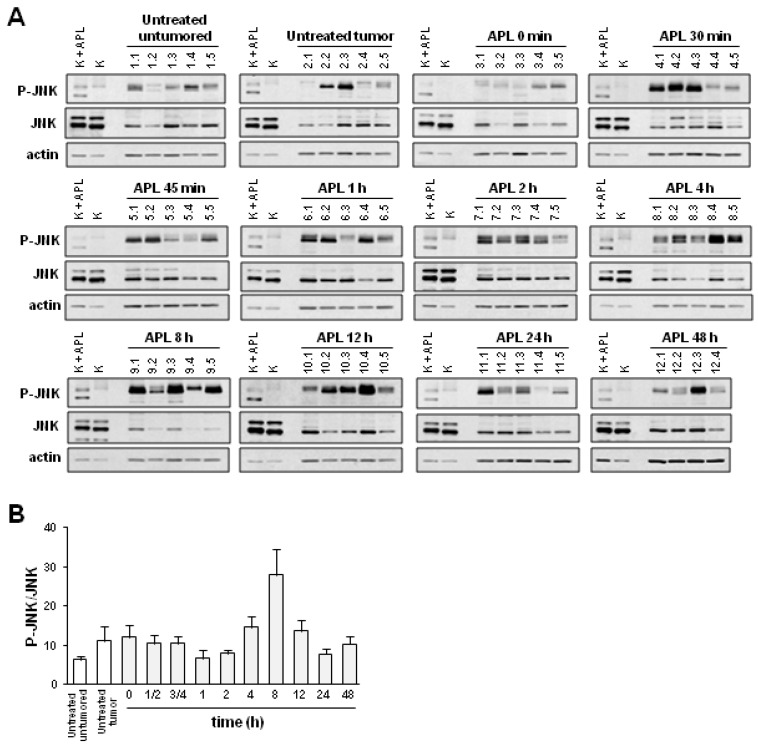
Plitidepsin increases the level of phosphorylated JNK in spleens of K562 tumor-bearing mice. Mice xenografted with K562 cells received a single administration of plitidepsin (APL; 200 μg/kg) and either immediately (0 min), or at the indicated times after injection, were sacrificed (5 mice/group). (**A**) Western blot analysis of total (JNK) and phosphorylated JNK (p-JNK) in spleens excised from the animals. Actin was used as loading control. K, K562 cells; K + APL, plitidepsin-treated K562 cells. (**B**) Quantification of p-JNK levels (normalized to those of total JNK) at the indicated times after APL administration and related to that of sample vehicle-treated K562 at the indicated times after APL administration. Mean ± SEM values of each group of animals are shown.

We then sought to compare phosphorylation of JNK in tumor tissues with that found in normal tissues that could be used as a surrogate for analyzing changes in the level of phosphorylated JNK in response to plitidepsin. For this purpose, we chose spleens that are a source of mononuclear cells. Interestingly, JNK phosphorylation increased in spleens from K562 tumor-bearing mice after plitidepsin administration (Figure 3A). Moreover, both tumor and spleen tissues responded similarly to plitidepsin treatment and there was a good correlation between the levels of phosphorylated JNK is each tissue type in each animal (Figure 3A). Consistent with previous experiments, densitometry analysis confirmed an increase in phosphorylated JNK in the spleens of mice at 4 to 12 h after treatment with plitidepsin, an effect that, as previously, was seen to reverse at 24 h post-treatment (Figure 3B). 

**Figure 4:**
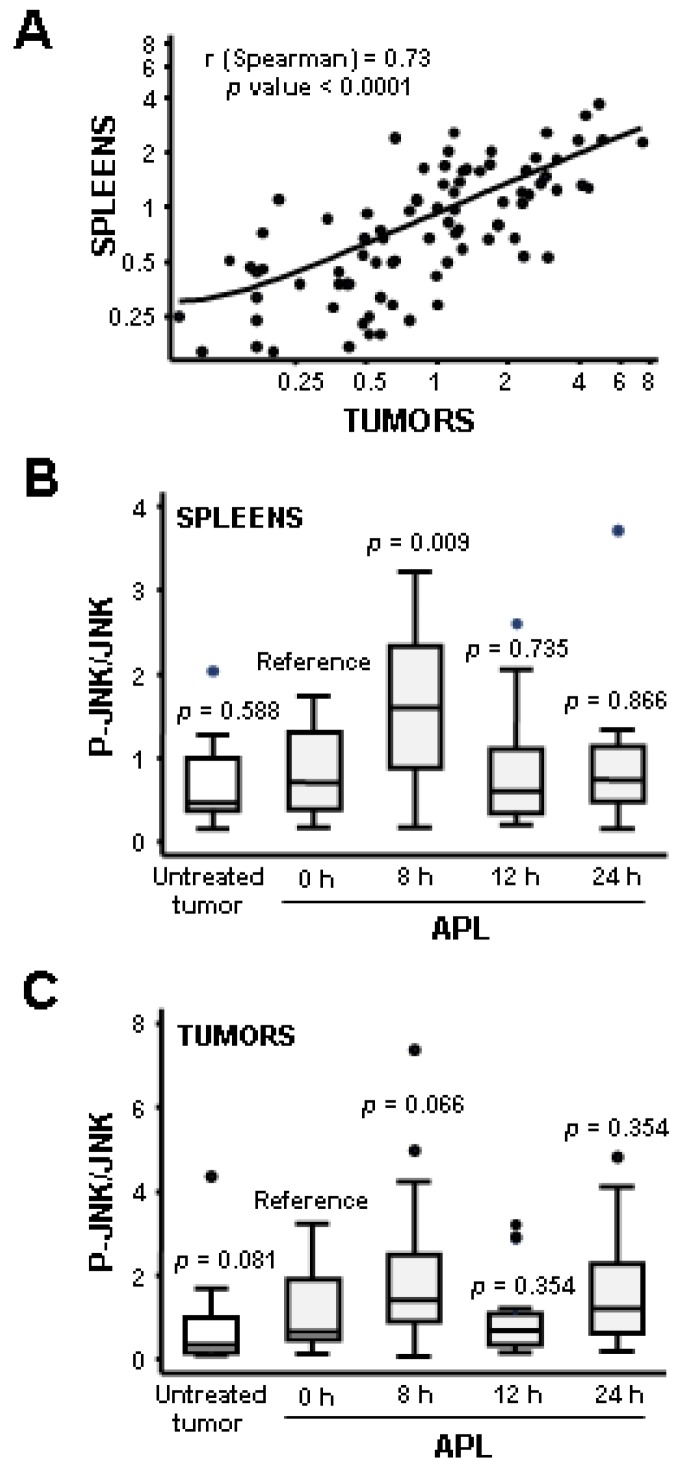
Statistical analysis of JNK activation by plitidepsin in tumors and spleens from mice xenografted with K562 leukemia cells. (**A**) Relation between the activation of JNK in tumors and spleens of K562 bearing-mice after plitidepsin administration. Samples corresponding to mice untreated and treated with plitidepsin (200 μg/kg) for 0, 8, 12 or 24 h were pooled. p-JNK/total JNK ratios in tumors and spleens were calculated and their correlation statistically assessed following a non-parametric approach. The Spearmans correlation coefficient (*r*) and *p* value are shown. (**B**, **C**) Box-plot of JNK activation in tumors and spleens of K562 bearing-mice after plitidepsin administration. The p-JNK/JNK ratio in spleens (**B**) and tumors (**C**) at each time point was compared to that of mice that were sacrificed just after plitidepsin injection (0 h) using Wilcoxon rank-sum test. A box-plot of JNK activation in each group (18–20 mice/group) and its statistical significance (*p*) are presented. Boxes in the plot include values in the 25%–75% interval; internal lines represent the median.

To confirm JNK phosphorylation as a pharmacodynamic biomarker of plitidepsin and to provide a better statistical analysis, we performed a new experiment using higher numbers of animals per group. Fifteen K562 tumor-bearing mice were treated with a single dose of plitidepsin (200 μg/kg) for 0, 8, 12, or 24 h, and the level of phosphorylated JNK was analyzed in both tumors and spleens by Western blot. To allow the signals in different membranes to be compared, samples from four untreated mice were also included in each group. Confirming earlier observations, a transient increase in JNK phosphorylation was found in response to plitidepsin in both tumors and spleens from xenografted mice (Supplementary Figure S5). In addition, and in order to include all data in the statistical analysis, samples from the first experiment were analyzed together with control samples from the second one (Supplementary Figure S6). For statistical evaluation, signals were quantified by densitometry and compared to control sample #64, which was selected as reference signal. A strong correlation was found between the increase in JNK phosphorylation in tumors and spleens of the same animal, with a Spearmans correlation coefficient of 0.73 and a *p* value <0.0001 (Figure 4A). In addition, a statistically significant increase in phosphorylated JNK was found at eight hours after plitidepsin treatment in spleens (*p* value = 0.009) (Figure 4B). In summary, these data demonstrated that JNK is activated in response to plitidepsin *in vivo* and suggest that JNK activation can be detected in both tumor and normal mouse tissues.

### 2.3. Plitidepsin Induces JNK Phosphorylation in Peripheral Mononuclear Blood Cells

**Figure 5:**
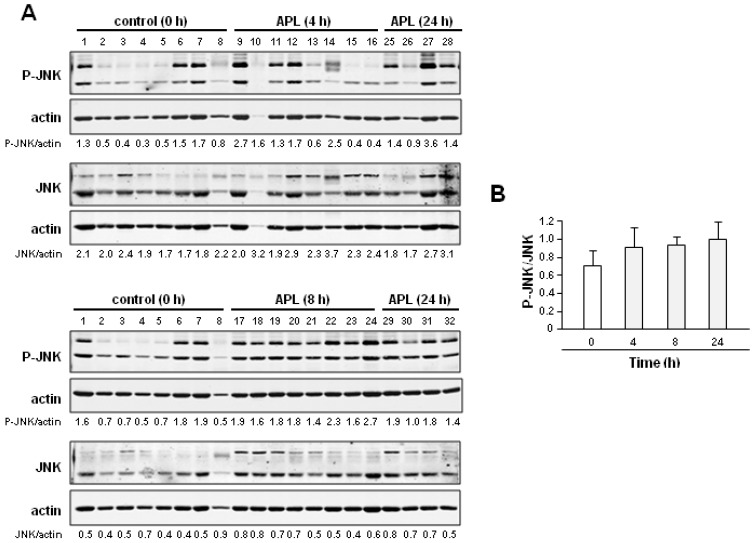
Plitidepsin increases JNK phosphorylation in rat peripheral mononuclear blood cells. (**A**) Blood mononuclear cells (PBMCs) were isolated from non-tumor-bearing rats after 4, 8 and 24 h i.v. administration of plitidepsin (1 mg/kg) and the levels of total (JNK) and phosphorylated JNK (p-JNK) were analyzed by Western blot. Control group were treated only with vehicle (control (0 h)). Actin was used as loading control for normalization. Immunoblots were incubated with infrared-labeled secondary antibodies, and the signals were visualized and quantified using the Odyssey Infrared Imaging System (Li-Cor Biosciences). (**B**) Quantification of the p-JNK/JNK ratio upon normalization to actin levels at the indicated times after plitidepsin administration in PBMCs derived from non-tumor-bearing rats. Mean ± SEM values of each group of animals are shown.

As the spleen is not an appropriate tissue in humans for regular pharmacodynamic biomarker measurement, we next determined whether plitidepsin could also affect JNK phosphorylation of mononuclear cells (PBMCs) in the peripheral blood of healthy animals. To this end, PBMCs were prepared from healthy rats following a single intravenous administration of plitidepsin (1 mg/kg), which yielded plasma concentrations of around 2 ng/mL and similar to those achieved in clinical trials [16,17] (Supplementary Table S1). As above, the levels of total and phosphorylated JNK were examined by Western blot. In order to improve quantification accuracy, we used the Odyssey Infrared Imaging System (Li-Cor Biosciences). In an initial time course analysis, we found a tendency for increased phosphorylated JNK in PBMCs at all post-treatment times studied (Figure 5).

**Figure 6:**
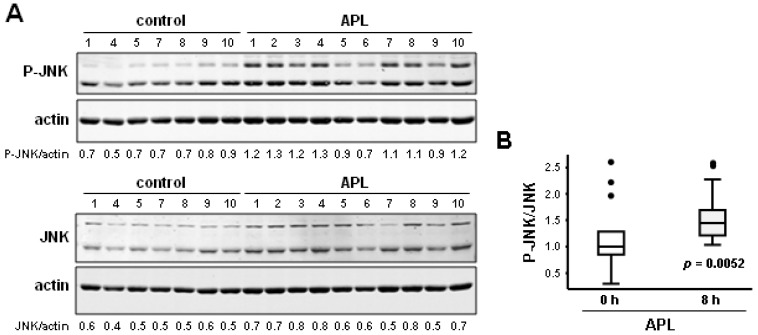
Plitidepsin induces a statistically significant increase of the level of phosphorylated JNK in the PBMCs of healthy rats after 8 h of administration. (**A**) Western blot analysis of JNK and p-JNK proteins in PBMCs obtained from rats treated with plitidepsin (1 mg/kg) for 8 h. Actin was used as loading control. Immunoblots were incubated with infrared-labeled secondary antibodies, and the signals were visualized and quantified using the Odyssey Infrared Imaging System (Li-Cor Biosciences). (**B**) Statistical analysis of JNK activation in PBMCs obtained from rats at 8 h after plitidepsin administration. Comparison between the p-JNK/JNK ratio in PBMCs of rats treated with plitidepsin or vehicle for 8 h using the Wilcoxon rank-sum test. A box-plot of JNK activation in each group (15–18 mice/group) and its statistical significance using Wilcoxon rank-sum test (*p*) are presented. Boxes in the plot include values in the 25%–75% interval; internal lines represent the median.

To allow a statistical analysis with a higher number of animals, we performed a new experiment at 8 h after plitidepsin administration. As shown in Figure 6A, plitidepsin increased the level of JNK phosphorylation in PBMCs at 8 h after administration. No significant changes in the level of total JNK protein were observed. Quantitative analysis using Odyssey software revealed a highly significant statistical increase in JNK phosphorylation (*p* = 0.005) in the PBMCs of rats 8 h after a single administration of plitidepsin (Figure 6B). This effect parallels the increase of JNK phosphorylation found in the tumor and spleen tissues of xenografted mice (Figure 4). Together, these data support the notion that phosphorylated JNK may serve as a pharmacodynamic marker for plitidepsin activity and suggest that changes in JNK activity in PBMCs could potentially be used as a biomarker when assessing plitidepsin treatment schedules in clinical trials.

### 2.4. Discussion

In this study we demonstrate that plitidepsin suppresses cell proliferation in a panel of hematological cancer cell lines with an IC_50_ in the nanomolar range, confirming its anti-tumor activity [1]. This growth inhibition is accompanied by a sustained activation of JNK that, together with data obtained in other cultured cells [3,4,5,9,11,15], led us to investigate JNK as a potential surrogate marker for plitidepsin activity. In agreement with this hypothesis, we found that plitidepsin does indeed increase the level of phosphorylated JNK *in vivo*, both in xenografted tumors generated using human leukemia K562 cells in immunodeficient mice as well as in the spleens of host animals. Importantly, the effect on JNK in tumors and spleens is parallel and concomitant. Although this does not necessarily mean that an equal effect on tumor-growth or toxicity against a surrogate tissue will be seen, it does show that the drug concentration used is appropriate to induce a robust biological response in the host animals.

Our data also shows that plitidepsin treatment at plasma concentrations comparable to those achieved in cancer patients increases JNK phosphorylation in PBMCs of healthy rats. This finding further suggests that normal tissues can be reliable markers of plitidepsin activity *in vivo*. This observation is particularly relevant since is not usually feasible to obtain tumor biopsies from patients, and furthermore the use of human PBMCs would allow sequential sampling over time for the assessment of different plitidepsin treatment schedules in a clinical setting. Our data showing that JNK activation upon plitidepsin administration might be used as an *in vivo* biomarker of response is consistent with the evidence that JNK is a protein critical for the anti-tumor activity of plitidepsin *in vitro* [6].

The mitogen-activated protein kinase pathway is a well accepted target for cancer therapy. Although JNK is implicated in oncogenic transformation and tumorigenesis due to its known ability to promote proliferation, a large number of studies have also linked JNK to tumor suppression. One mechanism of tumor suppression is mediated by the pro-apoptotic effects of JNK activation [18,19]. It seems that the extent and/or duration of activation of JNK is important in determining the cellular response; high or long activation leads to apoptosis, whereas low or transient activation leads to cell growth and differentiation [20]. Phosphorylation of JNK at residues (Thr^183^, Tyr^185^) that are recognized by the antibodies used in this study is linked to the activation of this enzyme and to the induction of apoptosis in many systems [21,22]. In cultured cells, sustained (hours) but not transient (minutes) activation of JNK, causes apoptosis in response to diverse stimuli that include anti-tumor drugs in clinical use [23,24]. Thus, cisplatin, 5-fluorouracil, etoposide, vinblastine and paclitaxel, among others, increase JNK phosphorylation in different human cancer cell types [25,26,27,28,29]. In our *in vivo* system, the increase in JNK phosphorylation was first detected a few hours after administration of plitidepsin to mice and lasted for at least 8 h before then declining to basal level. Although temporal correlations between *in vitro* and *in vivo* systems are difficult, such kinetics can be considered as a sustained activation of JNK. Notably, at least in human melanoma cells JNK mediates both dose-dependent plitidepsin-induced cell cycle arrest and apoptosis [3].

In contrast to JNK, the kinases p38MAPK and ERK, and the cell cycle inhibitor protein p27^KIP1^ that have been shown to be plitidepsin targets in several cultured cell types did not response to administration of the drug *in vivo* in these studies. This could be due to differences in cell specificity and/or to the *in vitro* conditions (culture media, plastic dishes, *etc.*). Alternatively or additionally, changes in p38MAPK, ERK and p27^KIP1^ by plitidepsin may require higher doses and/or longer times. 

Clearly, the identification of biomarkers of response in preclinical models is essential to the clinical development of novel drugs. In the clinic, biomarkers may help to identify the optimal dose and regimen, predict and monitor drug sensitivity, as well as allowing selection of patients most likely to respond to a particular therapeutic agent. Indeed, extensive preclinical biomarker studies have facilitated the development of numerous anticancer agents that have undergone clinical evaluation, including imatinib (Gleevev), trastuzumab (Herceptin), gefitinib (Iressa), sunitinib malate (Sutent), dasatinib (Sprycel) and everolimus (Afintor) [30,31,32,33,34,35,36,37]. Plitidepsin has successfully advanced to Phase II/III clinical trials for the treatment of hematological malignancies [38] (ADMYRE trial), but molecular targets that predict its response have not yet been identified. In this report, we show that plitidepsin suppresses the proliferation of cultured haematological cancer cells in parallel with a sustained activation of JNK, a protein associated with cancer. Importantly, plitidepsin induces the phosphorylation of JNK *in vivo* and this new finding supports the potential use of phosphorylated JNK as a pharmacodynamic biomarker to predict and monitor drug sensitivity in cancer patients. Furthermore, the results obtained show that phosphorylated JNK in spleens and in PBMCs is a reliable surrogate for phosphorylated JNK in tumors, and suggest that JNK activation by plitidepsin can be detected in normal tissues. Therefore, blood sampling could provide a source of material for biomarker evaluation of plitidepsin in the clinic. 

## 3. Experimental Section

### 3.1. Cell Culture and Drug Solutions

Human K562 chronic myelogenous leukemia, and RPMI8226, U266B1 and NCI-H929 multiple myeloma cells were obtained from the American Type Culture Collection (Manassas, VA, USA) and cultured in RPMI 1640 medium supplemented with 10% FCS and penicillin and streptomycin (all from GIBCO-Invitrogen, Paisley, UK). For tumor implantation, K562 cells obtained directly from the *in vitro* culture were suspended in 50% Matrigel^®^/RPMI 1640 media without serum or antibiotics. Passage 17 cells were implanted into the mice. Plitidepsin (Aplidin^®^, APL) was obtained from PharmaMar (Madrid, Spain). Stock solutions were freshly prepared in dimethylsulfoxide (DMSO). For *in vivo* studies, lyophilized vials of plitidepsin were reconstituted with vehicle (cremophor/ethanol/water) and further dissolved in PBS to reach the target dose.

### 3.2. Cell Proliferation Analysis

Cell proliferation was studied by [3-(4,5-dimethythiazol-2-yl)-2,5-diphenyl] tetrazolium bromide (MTT) assays that were performed following the manufacturer’s instructions (MTT Cell Proliferation Kit I, Roche Diagnostics, Mannheim, Germany).

### 3.3. Xenograft Tissues Studies

For the generation of K562 leukemia xenografts, we used 12 to 13 week-old (18–24 g) female athymic *nude* mice (Harlan, Madison, WI, USA). Mice were house in ventilated racks, provided with irradiated food and sterilized water *ad libitum*. On Day 1, mice were implanted with 5 × 10^6^ K562 cells per mouse subcutaneously. Tumor size measurements were recorded twice weekly beginning on Day 5 using vernier calipers. The formula to calculate volume for a prolate ellipsoid was used to estimate tumor volume from 2-dimentional tumor measurements: tumor volume (mm^3^) = (length × width^2^)/2. On Day 20, tumors reached an average volume of 532.8 mm^3^ ± 125.1 (mean ± SD) and mice were randomized into treatment and control groups. Treatments were initiated and administered on an individual body weight basis. Animals received a single bolus intraperitoneal dose of plitidepsin at 200 μg/kg and dosed at a volume of 10 mL/kg. Control groups were treated with vehicle alone. Then, at 0.5, 0.75, 1, 2, 4, 8, 12, 24, and 48 h post-dosing, mice were CO_2_ euthanized and exsanguinated via cardiac puncture. Tumors and spleens were harvested from each animal, individually identified and stored at −80 °C until further processed.

All animal protocols were reviewed and approved according to regional Institutional Animal Care and Use Committees.

### 3.4. Protein Lysates from Cells and *in Vivo* Tissues

Total protein extracts from cultured cell were prepared as described elsewhere [3,6]. To obtain protein lysates from mouse tissues (tumors and spleens) collected at various time points after dosing, samples were fragmented and pulverized in liquid nitrogen using a mortar. The resultant powder was transferred into a Potter-Elvehjem and homogenized with chilled lysis buffer (50 mM Tris-HCl (pH 7.5), 1% NP-40, 0.25% sodium deoxycholate, 150 mM NaCl, 2 mM MgCl_2_, 1 mM EDTA, 10% glycerol, 100 μg/mL PMSF, 10 μg/mL leupeptin, 10 μg/mL aprotinin, 30 nM GM6001 MMP inhibitor, 1 mM Na_3_VO_4_, 1 mM NaF, 10 mM β-glycerolphosphate and 0.5 mM DTT). Homogenates were centrifuged at 14,000× *g* at 4 °C for 20 min and the supernatants were transferred to new tubes. Protein extracts were quantified using the DC Protein Assay Kit (Bio-Rad, Hercules, CA, USA). To obtain peripheral blood mononuclear cells (PBMCs) from rats treated with plitidepsin, Sprague Dawley male rats (Harlan) received a single bolus dose of either plitidepsin (1 mg/kg) or placebo. Blood samples were collected at 4, 8 and 24 h post-dosing by cardiac puncture from deeply isofluorane-anesthetized animals. The blood was transferred into BD Vacutainer^®^ CPT Mononuclear Cell Preparation Tubes (Becton Dickinson, Heidelberg, Germany) and processed according to Manufacturer’s instructions. After the centrifugation (1800× *g*, 20 min, 25 °C), lymphocytes portion was isolated and cell pellets were resuspended in complete lysis buffer and processed for JNK expression as described for mouse tissues. The resulting plasma samples were aliquoted and frozen at −80 °C until bioanalysis. Plitidepsin quantification was performed by a sensitive LC/MS-MS method previously described [39].

### 3.5. Western Blot Analysis

Twenty μg protein were subjected to SDS-PAGE and transferred to PVDF membranes. Membranes were blocked at room temperature for 1 h in TBS-T (20 mM Tris pH 7.4, 136 mM NaCl, 0.1% Tween-20) containing 5% bovine albumin and incubated overnight at 4 °C with the appropriated antibody. Antibodies used were: anti-phosphorylated-JNK (sc-6254), anti-JNK (sc-474), anti-p38MAPK (sc-535), anti-phosphorylated-ERK (sc-7383), anti-ERK (sc-154) and anti-p27^KIP1^ (sc-776) from Santa Cruz Biotechnology, anti-phospho-JNK (#9251) and anti-phosphorylated-p38MAPK (#9211) from Cell Signaling and anti-c-Abl (Ab-3) from Calbiochem. Alexa-Fluor anti-mouse 680, Alexa-Fluor anti-rabbit 680 and Alexa-Fluor anti-goat 800 were from Li-Cor Bioscience. HPR-conjugated anti-mouse IgG (H + L) was from Promega, and HPR-conjugated anti-rabbit IgG (H + L) from MP Biomedicals. As a loading control, membranes were re-probed for actin (sc-1616) provided by Santa Cruz Biotechnology. After washing, blots were incubated with HPR-labeled or infrared-labeled secondary antibodies for 1 h at room temperature and developed by a peroxidase reaction using the ECL detection system (Amersham-G.E. Healthcare, Piscataway, NJ, USA) or visualized using the Odyssey Infrared Imaging System (Li-Cor Biosciences, Lincoln, NE, USA).

Immunoprecipitation of JNK was conducted by incubating 500 μg of protein extracts from tumors with 1 μg of the following antibodies: anti-JNK (sc-474) from Santa Cruz Biotechnology and anti-phosphorylated-JNK (#9251) from Cell Signaling, and collected on Gammabind-Sepharose beads (Amersham-Pharmacia Biotech, Piscataway, NJ, USA). Immunoprecipitated proteins were separated by SDS-PAGE and subjected to immunoblot analysis using anti-phosphorylated-JNK (#9251) from Cell Signaling or anti-phosphorylated-JNK (sc-6254) from Santa Cruz Biotechnology.

### 3.6. Statistical Analysis

Overall differences in JNK expression at different times in the same tissue were evaluated using Kruskal Wallis test. Two-by-two comparisons were also performed using Wilcoxon rank-sum test and taken the expression at time 0, that is, at the moment of plitidepsin injection, as reference. Correlation between the level of phosphorylated JNK measured in matched tumor and spleen samples from the same animal was assessed by computing the Spearman’s coefficient and its statistical significance. The data were analyzed by using STATA software (StataCorp LP, College Station, TX, USA).

## 4. Conclusions

Plitidepsin increases JNK phosphorylation in xenografted tumors and in surrogate rodent tissues. Our data provides evidence that phosphorylated-JNK may serve as a reliable marker for plitidepsin activity and suggests that changes in JNK activity of peripheral blood cells could be used as a pharmacodynamic marker for this agent.

## Supplementary Files

Supplementary File 1 Supplementary Material (PDF, 395 KB)
