# Supplementary material for: c-Jun N-Terminal Kinase Phosphorylation Is a Biomarker of Plitidepsin Activity
Source: Mar Drugs. 2013 May 21;11(5):1677–92. doi: 10.3390/md11051677 (PMC3707168; doi:10.3390/md11051677)
Supplement: Supplementary File 1 — Supplementary Material (PDF, 395 KB) [file marinedrugs-11-01677-s001.pdf]

Supplementary Material

Figure S1. Specificity of antibodies against phosphorylated JNK. JNK protein was immunoprecipitated from tumor extracts (#2.1 and #7.1; see Figure 2) using either anti-JNK antibody (sc-474, Santa Cruz; IP-1) or anti-p-JNK (#9251, Cell Signaling; IP-2). Two aliquots of each immunoprecipitate were subjected to SDS-PAGE followed by immunoblotting using anti-p-JNK sc-6254 antibody from Santa Cruz (panel **A**) or anti-p-JNK #9251 from Cell Signaling (panel **B**). For comparison, overexposed images are shown (low in both panels) to detect the signal in whole protein extracts.

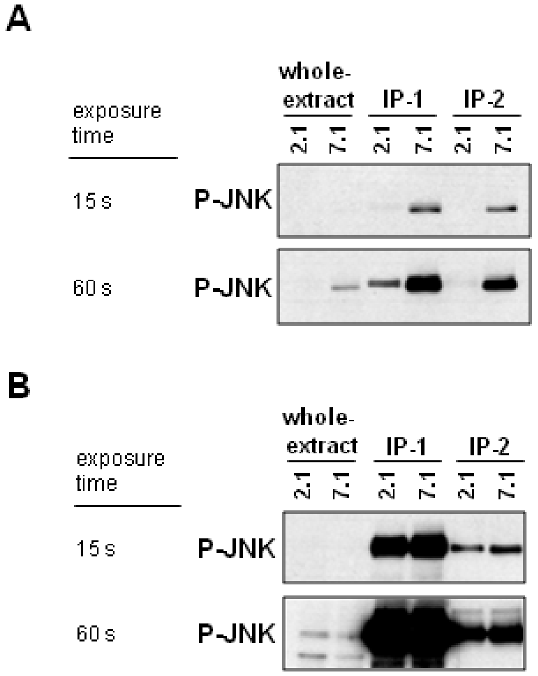

Figure S2. Levels of phosphorylated-p38MAPK and phosphorylated-ERK in xenografted tumors after plitidepsin treatment. K562 tumor-bearing mice were treated with plitidepsin (200 µg/kg) for the indicated times and tumors were excised, processed, and subjected to immunoblot analysis for total (p38) and phosphorylated p38MAPK protein (P-p38) and total (ERK) and phosphorylated ERK protein (P-ERK).

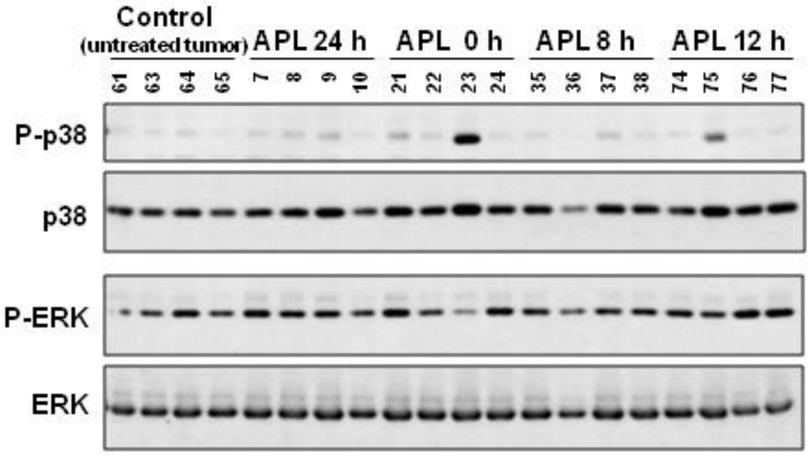

Figure S3. Levels of p27^{KIP1} in tumors of K562-bearing mice after plitidepsin treatment. K562 xenografted mice were treated with plitidepsin (200 µg/kg) for the indicated times and tumors were excised, processed, and subjected to immunoblot analysis for p27^{KIP1}. Actin was used as loading control.

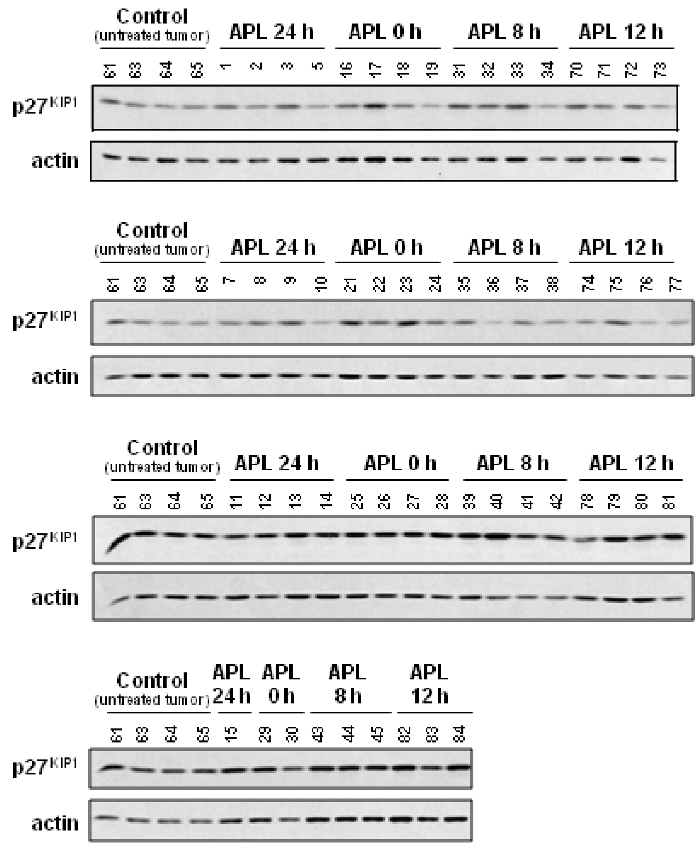

Figure S4. Levels of c-Abl and BCR-ABL proteins in tumors from mice xenografted with K562 cells. Western blot analysis of the expression of c-Abl and BCR-ABL (Bcr/Abl) proteins in tumors excised from mice xenografted with K562 cells that were treated with plitidepsin (200 µg/kg) for the indicated times. K, K562 cells; K + APL, plitidepsin-treated K562 cells.

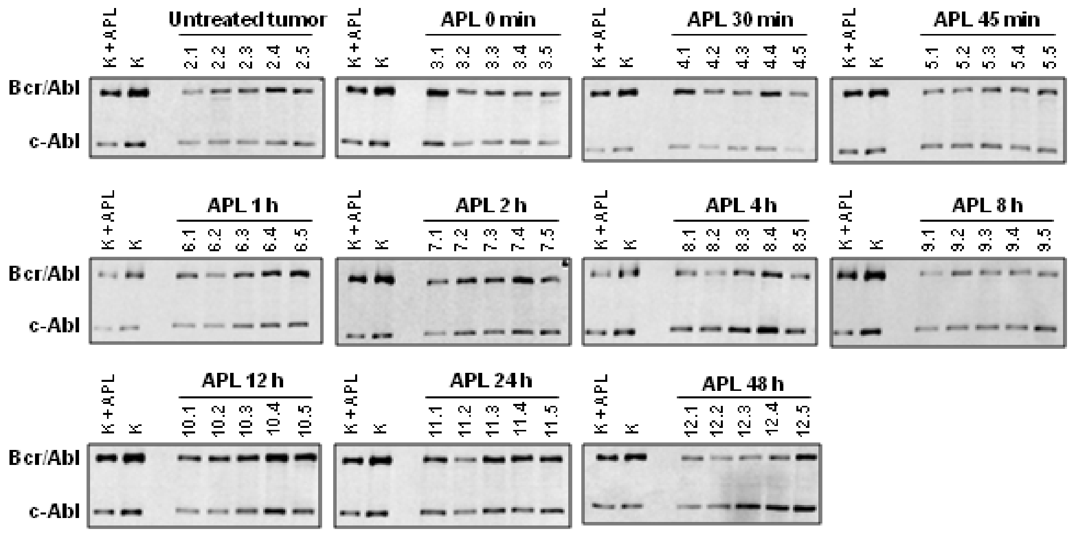

Figure S5. Levels of phosphorylated JNK in tumors and spleens of K562 tumor-bearing mice after plitidepsin treatment. Tumors (**A**) and spleens (**B**) from plitidepsin-treated xenografted mice (study #2) were excised, processed and subjected to Western blot analysis of total (JNK) and phosphorylated JNK (p-JNK). Actin was used as loading control. Values (Arbitrary Units, A.U.) correspond to p-JNK/JNK ratio in each sample normalized to actin and related to the same ratio in control sample #64.

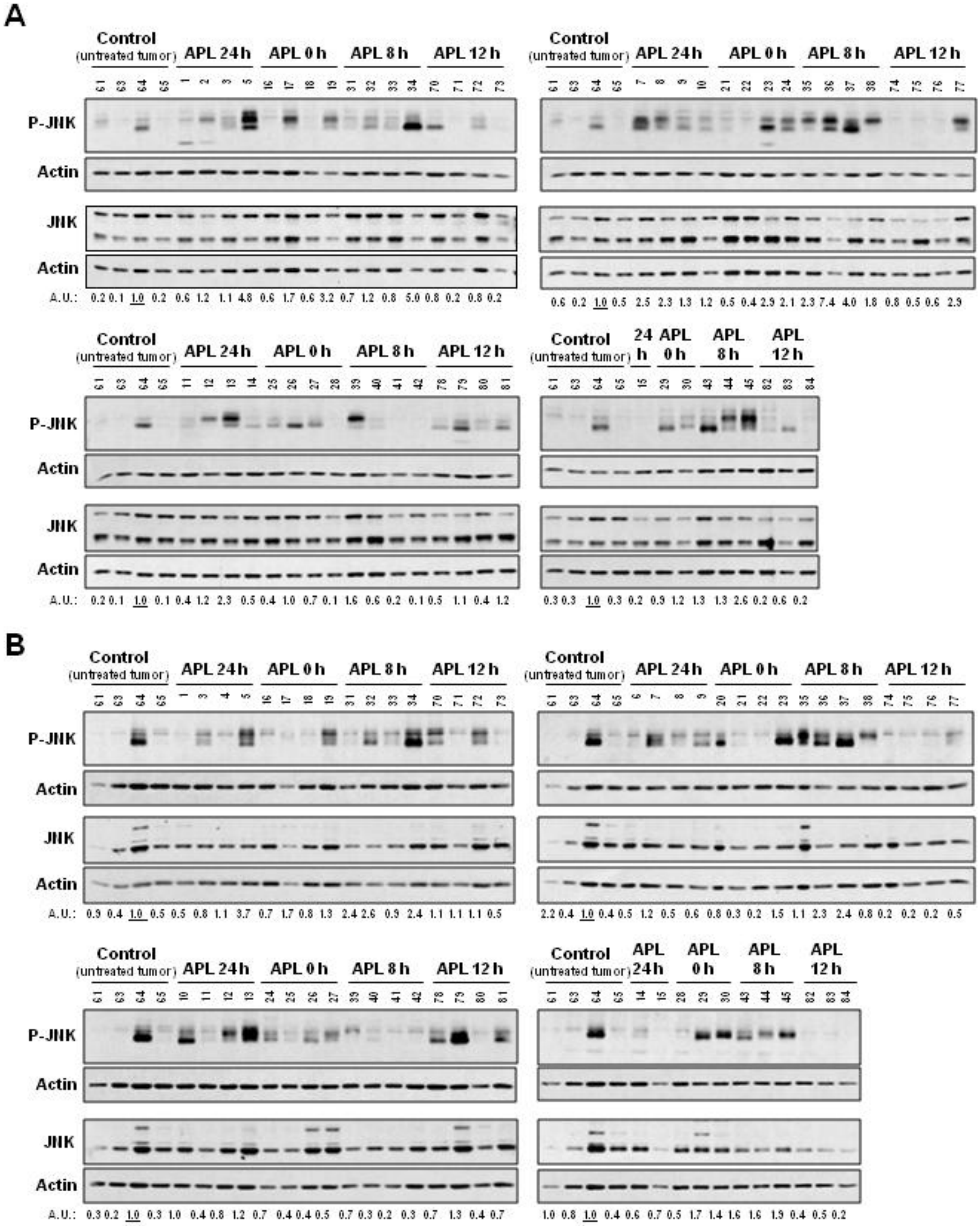

Figure S6. Levels of phosphorylated JNK in tumors and spleens from mice xenografted with human K562 leukemia cells after plitidepsin administration. Tumors (**A**) and spleens (**B**) from plitidepsin-treated xenografted mice (study #1; see Figure 2) were excised, processed and subjected to Western blot analysis of JNK and p-JNK together with control samples from study #2. Actin was used as loading control. Values (Arbitrary Units, A.U.) correspond to p-JNK/JNK ratio in each sample normalized to actin and related to the same ratio in control sample #64.

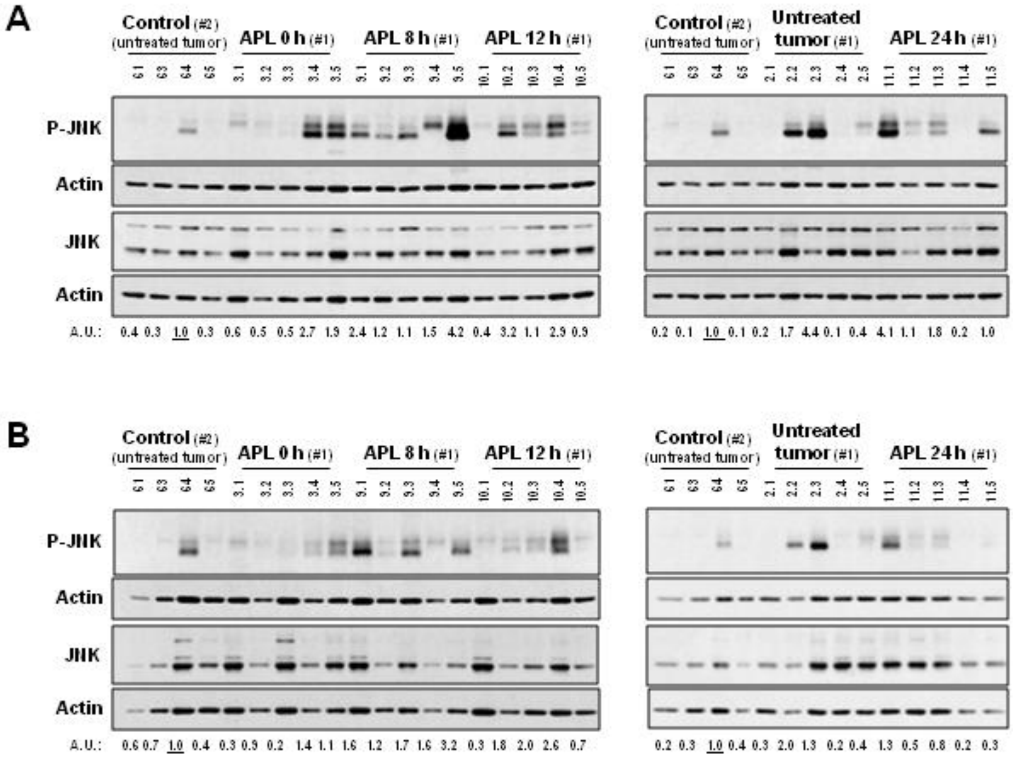

Table S1. Levels of plitidepsin in plasma from healthy rats after plitidepsin administration. Blood was isolated from non-tumor-bearing rats after 8 h i.v. administration of plitidepsin (1 mg/kg) (APL group; #APL1-10) or vehicle (control group; #C1-10), and the levels of plitidepsin in plasma were measured by a sensitive LC/MS-MS method [1]. Undetected; below limit of quantification, 0.05 ng/mL.

Animal (No.)	Treatment	Plasma [APL] (ng/mL)
#C1	Placebo	undetected
#C2	Placebo	undetected
#C3	Placebo	undetected
#C4	Placebo	undetected
#C5	Placebo	undetected
#C6	Placebo	undetected
#C7	Placebo	undetected
#C8	Placebo	undetected
#C9	Placebo	undetected
#C10	Placebo	undetected
#APL1	Plitidepsin	2.00
#APL2	Plitidepsin	1.47
#APL3	Plitidepsin	1.60
#APL4	Plitidepsin	1.71
#APL5	Plitidepsin	2.61
#APL6	Plitidepsin	2.50
#APL7	Plitidepsin	2.07
#APL8	Plitidepsin	2.22
#APL9	Plitidepsin	1.76
#APL10	Plitidepsin	3.76

References

1. Yin, J.; Aviles, P.; Lee, W.; Ly, C.; Floriano, P.; Ignacio, M.; Faircloth, G. Development of a liquid chromatography/tandem mass spectrometry assay for the quantification of aplidin, a novel marine-derived antineoplastic agent, in human plasma. *Rapid Commun. Mass Spectrom.* **2003**, *17*, 1909–1914.

© 2013 by the authors; licensee MDPI, Basel, Switzerland. This article is an open access article distributed under the terms and conditions of the Creative Commons Attribution license (http://creativecommons.org/licenses/by/3.0/).
